# Absence of Effect of Menopause Status at Initiation of First-Line Antiretroviral Therapy on Immunologic or Virologic Responses: A Cohort Study from Rio de Janeiro, Brazil

**DOI:** 10.1371/journal.pone.0089299

**Published:** 2014-02-20

**Authors:** Guilherme Amaral Calvet, Luciane Velasque, Paula Mendes Luz, Sandra Wagner Cardoso, Monica Derrico, Ronaldo Ismério Moreira, Angela Cristina Vasconcelos de Andrade, Andrea Cytryn, Elaine Pires, Valdiléa Gonçalves Veloso, Beatriz Grinsztejn, Ruth Khalili Friedman

**Affiliations:** 1 Instituto de Pesquisa Clínica Evandro Chagas (IPEC), Fundação Oswaldo Cruz, Rio de Janeiro, Brazil; 2 Departamento de Matemática e Estatística, Universidade Federal do Estado do Rio de Janeiro, Rio de Janeiro, Brazil; Salute San Raffaele University School of Medicine, Italy

## Abstract

**Objective:**

To compare the effectiveness of first-line combination antiretroviral therapy (cART) between premenopausal and postmenopausal women.

**Methods:**

ART-naïve women initiating cART between January 2000/June 2010 at the Instituto de Pesquisa Clínica Evandro Chagas Cohort were studied. Women were defined as postmenopausal after 12 consecutive months of amenorrhea. CD4 cell counts and HIV-1 RNA viral load (VL) measurements were compared between pre- and postmenopausal at 6, 12 and 24 months after cART initiation. Women who modified/discontinued a drug class or died due to an AIDS defining illness were classified as ART-failures. Variables were compared using Wilcoxon test, χ2 or Fisher’s exact test. The odds of cART effectiveness (VL<400 copies/mL and/or no need to change cART) were compared using logistic regression. Linear model was used to access relationship between CD4 change and menopause.

**Results:**

Among 383 women, 328 (85%) were premenopausal and 55 (15%) postmenopausal. Median pre cART CD4 counts were 231 and 208 cells/mm^3^ (*p = *0.14) in pre- and postmenopausal women, respectively. No difference in the median pre cART VL was found (both 4.8 copies/mL). Median CD4 changes were similar at 6 and 12 months. At 24 months after cART initiation, CD4 changes among postmenopausal women were significantly lower among premenopausal women (*p = *0.01). When the analysis was restricted to women with VL<400 copies/mL, no statistical difference was observed. Overall, 63.7% achieved cART effectiveness at 24 months without differences between groups at 6, 12 and 24 months.

**Conclusion:**

Menopause status at the time of first-line cART initiation does not impact CD4 cell changes at 24 months among women with a virologic response. No relationship between menopause status and virologic response was observed.

## Introduction

Women account for 50% of people living with HIV, the majority of them living in low and middle-income countries [1]. Worldwide, life expectancy has been increasing over the last several decades, even in developing countries, leading to a greater number of individuals older than 60 years. Brazil has one of the fastest aging populations in the world. In a half of a century (1960–2010), life expectancy of the Brazilian population increased by 25.4 years, having changed from 48.0 to 73.4 years [2].

The expansion of combination antiretroviral therapy (cART) coverage was crucial to reduce HIV-related morbidity and mortality rates turning HIV infection into a chronic condition. Antiretroviral therapy (ART) global coverage has significantly grown in the latest years, with 11.7 million life-years added to the world between 1996 and 2008 [3]. Consequently, the HIV/AIDS population is becoming older.

The number of older women who will become HIV-infected or who will live with HIV is expected to increase as overall life expectancy increases, and many of them will undergo menopause during the course of the HIV disease [4]. Prior to receiving antiretroviral therapy, younger HIV-infected women have higher CD4 cell counts and lower HIV RNA levels [5]–[7] when compared with HIV-infected men.

Recent studies have shown that aging has very specific effects on T cell function [8]. Natural sex steroids can mediate changes in the immune system and estrogens can regulate humoral and cellular immune responses with a decrease in CD4+ T and B lymphocytes subpopulations in postmenopausal women [9]–[11].

Several studies have evaluated the influence of gender and age on cART outcomes [12]–[15]. cART effectiveness in women may be different according to menopausal status, and previous studies have shown conflicting results [16], [17].

Data on the cART outcomes in HIV-infected postmenopausal women remain scarce, especially in low- and middle-income settings, including Brazil, where universal access to cART free of cost has been provided by the Ministry of Heath since 1997. The purpose of this study was to compare the effectiveness of first-line cART among HIV-infected pre- and postmenopausal women in a cohort of HIV-infected women in Rio de Janeiro, Brazil.

## Methods

### Ethical Statement

The study protocol was reviewed and approved by the ethics committee of Evandro Chagas Clinical Research Institute, Oswaldo Cruz Foundation (CAE 0032.0.009.000-10). Written informed consent was obtained from all women.

### Description of the Cohort and Study Population

This study was conducted at the Instituto de Pesquisa Clínica Evandro Chagas (IPEC) AIDS Service at Oswaldo Cruz Foundation (Fiocruz), Rio de Janeiro, Brazil, where care has been provided to HIV/AIDS patients since 1986. An observational, longitudinal, clinical database is maintained on patients receiving primary and specialized HIV care at the clinic. Details of the HIV/AIDS cohort can be found elsewhere [18], [19]. To study the natural history of HIV infection in women, a prospective open cohort was established at IPEC in 1996. Study visits occur every 6 months; sociodemographic, behavior, reproductive, gynecologic and laboratory data are collected using structured questionnaires [20], [21].

For this study, data from 386 antiretroviral-naïve women who initiated cART between January 1, 2000 and June 30, 2010 were considered. Women without gynecological data defining menopause status were excluded (N = 3). Thus, we analyzed data from 383 cART-naïve patients who initiated cART within the study period. Follow up information includes data up to September 30, 2011.

### Study Definitions

Menopausal status was prospectively captured during bi-anual interviews. Women were defined as postmenopausal after twelve consecutive months of amenorrhea, for which there was no other obvious pathological or physiological cause [22]. Women with a history of hysterectomy were only considered postmenopausal if they had undergone bilateral oophorectomy regardless of age. All other women were classified as premenopausal.

Race was based on provider report and categorized as white and nonwhite.

cART was defined as two nucleosides transcriptase reverse inhibitors (NTRI) in combination with one non nucleoside transcriptase reverse inhibitor (NNRTI) or one protease inhibitor (PI); antiretroviral regimens were classified as NNRTI or PI-based. The calendar year of cART initiation was stratified into two groups: 2000–04 and 2005–09.

Drug class modifications and discontinuation were defined as any NNRTI or PI drug modification or interruption (including deaths due to AIDS defining illnesses). Neither NTRI substitutions nor regimen dosage adjustments were considered regimen modifications.

The effectiveness of first-line cART at 6, 12 and 24 months was defined as an HIV-1 RNA viral load (VL) measurement of less than 400 copies/mL at these time points without drug class modification. Window periods were defined for each time point as 5–9 months, 9–15 months, and 21–27 months, respectively. Within each window, the viral load and CD4 cell counts (cells/mm^3^) measurement recorded closest to the time points were evaluated.

Baseline CD4+ lymphocyte counts was defined as the value obtained within 90 days of cART initiation (before or after) and baseline HIV-1 RNA as any available value obtained before or up to 7 days after cART initiation. CD4+ cell counts and HIV-1 RNA results were obtained from the medical records. CD4+ cell counts were categorized into <200, 200–500 and >500 cells/mm^3^. Plasma HIV VL was categorized into 401–10,000, 10,001–100.000 and >100,000 copies/mL.

CD4+ T-cell counts samples were evaluated on a BD FACSCalibur cytometer (Becton Dickinson, USA). The assays used for measurement of HIV-1 viral load were nucleic acid sequence–based amplification (NASBA, Organon Teknika, Boxtel, The Netherlands), Roche Amplicor reverse transcriptase polymerase chain reaction (RT-PCR) assay (Roche Molecular Diagnostics, USA) and branched DNA assay, Versant HIV-1 RNA 3.0 (Siemens, Tarrytown, USA). The assays used for measurement of HIV-1 viral load varied according to the year and whether or not the woman was enrolled in a clinical trial. For the purpose of statistical analysis, the cut-off value, defined as undetectable VL, was set as a VL ≤400 copies/mL, regardless of the method used.

AIDS defining illnesses (ADI) was defined as the presence of any 1993 Centers for Disease Control and Prevention (CDC)-defined ADI [23] at 90 days prior up to 30 days after cART initiation baseline.

Participation in a clinical trial was defined when a patient started their first-line regimen in a cART-naïve clinical trial.

### Statistical Analysis

Quantitative variables between pre- and postmenopausal women were compared using Wilcoxon test. Categorical variables were compared using chi*-*squared test or Fisher’s exact test. Median changes in CD4 cell counts and HIV-1 RNA VL measurements were compared between pre- and postmenopausal women at 6, 12 and 24 months after cART initiation.

We calculated the odds ratio for effective cART and the 95% confidence interval (95%CI) of first-line cART at 6, 12 and 24 months after treatment initiation, adjusting for baseline log 10 VL, baseline CD4, cART regimen and ADI, using logistic regression model.

The impact of missing VL measurement on the effectiveness of first-line cART was evaluated in a sensitivity analyses. We compared the estimates and confidence intervals of three different models. 1) Model A: when individuals with missing VL measurements were excluded; 2) Model B: when the missing VL measurements were coded as 1 (missing values assumed to be failure yielding a worse-case scenario) and; 3) Model C: when the missing VL measurements were coded 0 (missing values assumed to be effective yielding a best-case scenario).

Linear regression was used to access the relationship between CD4 change and menopause at 6, 12 and 24 months. In the other analyses, immunological reconstitution was defined as a >25% increase in CD4 cell count from baseline and logistic regression was used to access the menopause effect at 6, 12 and 24 months. All models were adjusted for baseline log 10 VL, baseline CD4, cART regimen and ADI.

For all statistical analyses we used the statistical software R, version 2.14.2 (www.r-project.org).

## Results

Among 383 women, 328 (85%) were premenopausal and 55 (15%) postmenopausal. Demographic and clinical characteristics are shown in table 1. There were no significant differences in characteristics between the two subgroups, except for age and history of hysterectomy. The median age was 34 years [interquartile interval (IQR): 28–40] for premenopausal and 52 years (IQR: 48–55) for postmenopausal women (*P*<.001). A hysterectomy history was more frequent among postmenopausal women (18.2% vs. 1.2% in premenopausal women, p<0.001).

**Table 1:** Demographic and clinical characteristics for premenopausal and postmenopausal women at the start of antiretroviral therapy (baseline).

Characteristic		Premenopause(N = 328)	Postmenopause(N = 55)	Total(N = 383)	p-value
Age, median years (IQR)		34 (28–40)	52 (48–55)	36 (30–43)	<0.001
Race/ethnicity, N (%)	White	136 (41.5)	28 (50.9)	164 (42.8)	0.239
	Non-white	192 (58.5)	27 (49.1)	219 (57.2)	
Hysterectomy, N (%)		4 (1.2)	10 (18.2)	14 (3.6)	<0.001
Baseline CD4 cell count, median (IQR, cells/mm^3^)		231(132–334)	208 (85–287)	227(127–329)	0.14
Baseline CD4 cell count (cells/mm^3^), N (%)	<200	112 (40.3)	24 (49.0)	136 (41.6)	0.495
	200–500	149 (53.6)	23 (46.9)	172 (52.6)	
	>500	17(6.1)	2 (4.1)	19 (5.8)	
Baseline HIV viral load, median (IQR, log 10 copies/mL)		4.8 (4.1–5.4)	4.8 (4.4–5.3)	4.8 (4.2–5.4)	0.18
Baseline HIV viral load (copies/mL), N (%)	401–10,000	51 (20.4)	3 (6.5)	54 (18.2)	0.071
	10,001–100,000	98 (39.2)	23 (50.0)	121 (40.9)	
	>100,000	101 (40.4)	20 (43.5)	121 (40.9)	
AIDS defining illness, N (%)	Yes	68 (20.7)	13 (23.6)	81 (21.2)	0.597
cART regimen, N (%)	PI	91 (27.7)	18 (32.7)	109 (28.5)	0.518
	NNRTI	237 (72.3)	37 (67.3)	274 (71.5)	
Year of starting cART, N (%)	2000–2004	118 (36.0)	21 (38.2)	139 (36.3)	0.764
	2005–2009	210 (64.0)	34 (61.8)	244 (63.7)	
In clinical trial, N (%)	Yes	125 (38.1)	20 (36.4)	145 (37.9)	0.881

IQR, interquartile interval;

cART: Combination Antiretroviral Therapy; PI: Protease inhibitor;

NNRTI: Non-Nucleoside Reverse Transcriptase Inhibitors.

Median pre-cART CD4 counts were 231 (IQR:132–334) and 208 (IQR:85–287) cells/mm3 (*p* = 0.14) in pre- and postmenopausal women, respectively. No difference in median pre cART VL was observed (both 4.8 copies/mL). Almost two- thirds of the study population initiated cART between 2005 and 2009. NNRTI-based cART was the most frequently prescribed (72.3% and 67.3% in pre- and post-menopausal women, respectively). Approximately 40% of all women initiated cART within a clinical trial.

The most frequent first cART regimens stratified by menopausal status are depicted in Table 2. A combination of zidovudine (ZDV)+lamivudine (3TC)+efavirenz (EFV) was used by almost two-fifths of the study population (148, 38.6%).

**Table 2:** Most frequent first cART regimens stratified by menopausal status at the initiation of antiretroviral therapy (baseline).

First cART regimens	Premenopause N = 328 (%)	Postmenopause N = 55 (%)	Total N = 383 (%)
ZDV+3TC+EFV	123 (37.5)	25 (45.4)	148 (38.6)
FTC+TDF+EFV	42 (12.8)	5 (9.1)	47 (12.3)
TDF+3TC+EFV	27 (8.2)	4 (7.3)	31(8.1)
d4T+3TC+EFV	12 (3.7)	3 (5.5)	15 (3.9)
ZDV+3TC+ATV	14 (4.3)	1 (1.8)	15 (3.9)
FTC+ddI+ATV	10 (3.1)	3 (5.5)	13 (3.4)
ZDV+3TC+NFV	11 (3.4)	2 (3.6)	13 (3.4)
ZDV+3TC+LOP/r	9 (2.7)	3 (5.5)	12 (3.1)
TDF+3TC+ATV/r	8 (2.4)	1 (1.8)	9 (2.3)
d4T+3TC+NFV	4 (1.2)	3 (5.5)	7 (1.8)
ZDV+3TC+ATV/r	6 (1.8)	1 (1.8)	7 (1.8)
ZDV+3TC+SQV/r	5 (1.5)	1 (1.8)	6 (1.6)
ZDV+3TC+IDV/r	2 (0.6)	1 (1.8)	3 (0.8)
TDF+3TC+LOP/r	1 (0.3)	2 (3.6)	3 (0.8)
Other NNRTI-based cART	33 (10.1)	–	33 (8.6)
Other PI-based cART	21 (6.4)	–	21 (5.5)

3TC, lamivudine; ATV/r, atazanavir/ritonavir; d4T, stavudine; ddI, didanosine; EFV, efavirenz;

FTC, emtricitabine; IDV/r, indinavir/ritonavir; LOP/r, lopinavir/ritonavir; SQV/r, saquinavir/ritonavir;

NFV, nelfinavir; TDF, tenofovir; ZDV, zidovudine;

cART: combination antiretroviral therapy; PI: Protease inhibitor;

NNRTI: Non-Nucleoside Reverse Transcriptase Inhibitors.

CD4 count median changes were similar in pre- and postmenopausal women at 6 (101 vs. 106 cells/mm^3^; *p* = 0.73) and 12 months (171 vs. 147 cells/mm^3^; *p* = 0.42). At 24 months after cART initiation, CD4 median changes among postmenopausal women were significantly lower than among premenopausal women (184 vs. 273 cells/mm^3^; respectively; *p* = 0.02) (table 3).

**Table 3:** cART effectiveness at 6, 12, and 24 months and the median change in CD4 cell count for premenopausal and postmenopausal antiretroviral-naïve women.

Characteristic	Time points	N	Premenopause	Postmenopause	total	p-value
**All patients**						
cART effectiveness, N (%)	6 months	309	189 (71.3)	32 (72.7)	221 (71.4)	0.99
	12 months	310	189 (71.3)	33 (73.3)	223 (71.2)	0.94
	24 months	293	164 (64.3)	24 (60.5)	188 (63.7)	0.60
Change CD4, median (IQR, cells/mm^3^)	6 months	260	101 (31–193)	106 (69–201)	101 (31–194)	0.73
	12 months	273	171 (76–290)	147 (75–278)	163 (72–285)	0.42
	24 months	251	273 (156–395)	184 (116–282)	262 (149–384)	**0.02**
>25% CD4 cell count increase [N (%)]	6 months	260	145 (65.3)	27 (71.1)	172 (66.2)	0.61
	12 months	273	175 (75.4)	31 (75.6)	206 (75.5)	0.86
	24 months	251	187 (86.6)	28 (80.0)	215 (85.7)	0.44
**In patients with viral load ≤400 copies/mL**						
Change CD4, median (IQR, cells/mm^3^)	6 months	200	119 (56–204)	99 (60–199)	113 (56–202)	0.79
	12 months	207	182 (106–300)	152 (52–268)	179 (97–299)	0.20
	24 months	204	298 (188–439)	244 (154–396)	291 (178–432)	0.27
>25% CD4 cell count increase [N (%)]	6 months	200	122 (70.9)	19 (67.9)	141 (70.5)	0.92
	12 months	207	143 (80.8)	21 (70.0)	164 (79.2)	0.27
	24 months	204	161 (90.4)	23 (88.5)	184 (90.2)	0.73

IQR, interquartile interval;

cART: Combination Antiretroviral Therapy;

When analysis was restricted to women with VL<400 copies/mL in both groups, no statistical differences were observed between pre- and postmenopausal, although CD4 median changes were lower among postmenopausal women at all-time points (6, 12 and 24 months) (table 3). No differences were found between the proportions of pre- and postmenopausal women who achieved VL<400 copies/mL at 6 (71.3% vs. 72.7%, respectively; *p = *0.99), 12 (71.3% vs. 73.3%, respectively; *p = *0.94), and 24 months (64.3% vs. 60.5%, respectively; *p = *0.60) (table 3).

There were no differences in the odds ratio of achieving an HIV-1 RNA level <400 copies/mL for premenopausal compared with postmenopausal women after adjusting for baseline log10 HIV-1 RNA levels, baseline CD4, cART regimen and ADI at 6 months (OR = 1.00; 95% CI: 0.86–1.17), 12 months (OR = 1.02; 95% CI: 0.87–1.19), and 24 months (OR = 1.02; 95% CI: 0.85–1.22) (table 4). Sensitivity analyses performed to assess the impact of missing information in the evaluation of cART effectiveness did not show potential selection bias. In this analysis (data not shown), the estimates of all the variables retained in the final model A did not differ significantly (overlap 95% CI) from the ones estimated in models B and C.

**Table 4:** cART effectiveness and CD4 change models at 6, 12, and 24 months for premenopausal and postmenopausal antiretroviral-naïve women.

	6 months	12 months	24 months
**All patients**	N = 230	N = 228	N = 210
cART effectiveness [OR (95%CI)]	1.00 (0.86–1.17)	1.02 (0.87–1.19)	1.02 (0.85–1.22)
	N = 213	N = 222	N = 207
CD4 Change coefficients (Standard error)	−7.6(29.0)	−27.4(31.2)	**−97.8(39.9)**
	N = 213	N = 222	N = 207
>25% CD4 cell count increase [OR(95%CI)]	1.06 (0.43–2.59)	0.69 (0.29–1.69)	0.46 (0.17–1.26)
**In patients with viral load ≤400 copies/mL**	N = 164	N = 167	N = 166
CD4 Change coefficients (Standard error)	−14.3(34.8)	−35.4(35.9)	−61.1(44.4)
	N = 164	N = 167	N = 166
>25% CD4 cell count increase [OR(95%CI)]	0.69 (0.24–1.95)	0.38 (0.14–1.07)	0.46 (0.11–1.92)

cART: Combination Antiretroviral Therapy; OR: Odds Ratio.

Models adjusted for baseline log 10 HIV-1 RNA levels, baseline CD4, HAART regimen and AIDS defining illness.

p<0.05 in bold.

At 24 months after cART initiation (and after adjusting for the same covariates), postmenopausal women had significantly lower CD4 cell changes (−97.8 cells/mm^3^; SE: 39.9 cells/mm^3^) than premenopausal women (*p = *0.01). This difference was not observed when analysis was restricted to women with VL<400 copies (−61cells/mm^3^; SE: 44.4 cells/mm^3^) (p = 0.26) (table 4).

When immunological reconstitution was evaluated as a >25% increase in CD4 cell count from baseline, no statistical differences were observed in unadjusted or adjusted analyses comparing pre- and postmenopausal women at 6, 12 and 24 months after cART initiation in the overall group or when the analysis was restricted to virologically suppressed women (tables 3 and 4).

## Discussion

Our results demonstrated that menopause status at the time of initiation of first-line combination antiretroviral therapy does not impact CD4 cell count changes at 24 months among women with virologic response, regardless of the ART class prescribed, year of ART initiation or having started ART in a clinical trial as compared to regular care. Nevertheless, CD4 median changes remained lower among postmenopausal women.

We found no evidence of any interaction between menopause and virologic responses. To our knowledge, our analysis is the first to report virologic and immunologic outcomes following cART initiation in women from a middle-income country stratified by menopause status.

Most cohort studies found that overall, older people can achieve treatment success rates similar to younger individuals [24], [25]. Some investigators indirectly evaluated the impact of reproductive hormones levels on CD4 lymphocyte counts [17], [26]. In a study from the Swiss cohort, postmenopausal women had lower CD4 lymphocyte counts 3 years after seroconversion as compared to premenopausal women, although the differences were not statistically significant [17]. A meta-analysis involving 4414 antiretroviral-naïve women enrolled in 32 randomized trials between 2000 and 2010 concluded that women aged 50 or older had significantly greater chances of viral suppression in response to a first-line cART regimen than women 35 or younger at weeks 24 and 48. No significant age differences on immunologic responses at weeks 24 or 48 were observed, although younger women taking a NNRTI based cART regimen had significantly better CD4-cell responses than the older group through 48 weeks of treatment [26]. Treatment compliance may explain these outcomes because older patients have been found to be more adherent to HIV medications than younger patients [27]–[29].

Several studies have shown a decreased CD4 cell response among older patients, regardless of sex, and some of them have observed increased HIV disease progression and a negative impact on survival [14], [15], [30]–[34]. Among 24107 HIV-infected adults enrolled in the International epidemiological Database to Evaluate AIDS (IeDEA) Collaboration in the West African region, a significantly higher mean CD4 gain was observed among younger patients when compared to elderly patients after 12 months of cART initiation [15]. A study from the French Hospital Database including 3015 antiretroviral-naïve patients found that patients over 50 years old had a significantly slower CD4 cell reconstitution and a significantly higher risk of clinical progression than younger patients, despite a better virologic response [30]. Althoff et al in a pooled analysis of 19 prospective cohort studies in the North American AIDS Cohort Collaboration on Research and Design (NA-ACCORD) showed that the immunologic response decreased with increasing age after 24 months of initial cART regimen, regardless of ART class and that older individuals were less likely to have a CD4 increase greater than 100 cells [31].

Abrogoua et al, in a study conducted in a resource-limited setting, found that age and baseline clinical status had no significant influence on immunological outcomes at 24 months, although an optimal CD4 cell response was significantly influenced by adherence [29]. We were unable to evaluate the impact of adherence on virological and immunological outcomes in our study population as adherence data were not available.

Paterson et al, in an analysis comparing the effect of sex by age strata, did not find differences at 6 months from the initiation of a first HAART regimen among previously ART naïve patients in either immunological reconstitution or virological response. The potency of a first HAART regimen in controlling HIV-1 replication and subsequent immunological reconstitution have probably been able to mask any more subtle effect of biological sex differences on treatment responses [35]. In a subsequent study evaluating long-term immunologic and virologic responses to initial ART in pre- and post-menopausal women participating in two treatment trials similar virologic and immunologic responses to ART in treatment naïve pre- and post-menopausal women initiating ART in a clinical trial setting were observed [16].

Our results are reassuring that women can be treated similarly irrespective of age. Our analysis, however, did not include evaluations of antiretroviral-related toxicities or other adverse events which have been reported to be increased among older individuals and women including in our cohort [18], [27], [36], [37]. Despite this, 73.3% of the post-menopausal women and 71.3% of premenopausal women with HIV-1 RNA measurements were virologically suppressed at 24 months.

Strengths of this study include a large urban cohort of women receiving HIV clinical care coupled with specialized gynecological care with prospective standardized bi-annual data collection on reproductive health, which reduces the risk of recall bias in regards to age at menopause.

Limitations of this study include the retrospective nature of the HIV clinical and laboratory data collection, and a certain level of missing data on CD4 and viral load, although sensitivity analysis did not show potential selection bias. Moreover, the lack of adherence data precluded a systematic adherence evaluation.

In summary, in our study population, menopause status at cART initiation did not impact long-term immunological outcomes in women with favorable virologic responses. We found no evidence of an interaction between menopause status and virologic responses. These results are reassuring, given the increasing number of older HIV-infected women initiating cART and the impact of immune reconstitution on long-term survival.
